# Systemic oxidative stress in children with cystic fibrosis with bacterial infection including *Pseudomonas Aeruginosa*

**DOI:** 10.1111/crj.13513

**Published:** 2022-06-26

**Authors:** Gabriela Datsch Bennemann, Emilia Addison Machado Moreira, Leticia Cristina Radin Pereira, Maiara Brusco de Freitas, Diane de Oliveira, Julia Carvalho Ventura, Eduardo Benedetti Parisotto, Yara Maria Franco Moreno, Erasmo Benicio Santos Moraes Trindade, Eliana Barbosa, Norberto Ludwig Neto, Danilo Wilhelm Filho

**Affiliations:** ^1^ Graduate Program in Nutrition Federal University of Santa Catarina Florianópolis Brazil; ^2^ Department of Community Health Sciences, Cumming School of Medicine University of Calgary Calgary AB Canada; ^3^ School of Pharmaceutical Sciences, Food and Nutrition Federal University of Mato Grosso do Sul Campo Grande Brazil; ^4^ Department of Nutrition, Graduate Program in Nutrition Federal University of Santa Catarina Florianópolis Brazil; ^5^ Joana de Gusmão Children's Hospital Government of Santa Catarina Florianópolis Brazil; ^6^ Department of Ecology and Zoology, Graduate Program in Pharmacy Federal University of Santa Catarina Florianópolis Brazil

**Keywords:** antioxidants, bacterial infection, children, cystic fibrosis, oxidative stress

## Abstract

**Introduction:**

Oxidative stress (OS) occurs in cystic fibrosis (CF).

**Objective:**

The objective of this work is to evaluate the influence of bacterial infection on biomarkers of OS (catalase [CAT], glutathione peroxidade [GPx], reduced glutathione [GSH]), markers of oxidative damage (protein carbonyls [PC], thiobarbituric acid reactive substances [TBARS]), together with the nutritional status and lung function in children with CF.

**Methods:**

Cross‐sectional study including CF group (CFG, *n* = 55) and control group (CG, *n* = 31), median age: 3.89 and 4.62 years, respectively. CFG was distributed into CFG negative bacteriology (CFGB−, *n* = 27) or CFG positive bacteriology (CFGB+, *n* = 28), and CFG negative *Pseudomonas aeruginosa* (CFGPa−, *n* = 36) or CFG positive *Pseudomonas aeruginosa* (CFGPa+, *n* = 19).

**Results:**

Compared with CG, CFG (*P* = .034) and CFGB+ (*P* = .042) had lower body mass index‐for‐age *z*‐score; forced expiratory volume in the first second was lower in CFGB+ and CFGPa+ (both *P* < .001). After adjusting for confounders and compared with CG: CFG showed higher TBARS (*P* ≤ .001) and PC (*P* = .048), and lower CAT (*P* = .004) and GPx (*P* = .003); the increase in PC levels was observed in CFGB+ (*P* = .011) and CFGPa+ (*P* = .001) but not in CFGB− (*P* = .510) and CFGPa− (*P* = .460).

**Conclusions:**

These results indicate a systemic OS in children with CF. The presence of bacterial infection particularly *Pseudomonas aeruginosa* seems to be determinant to exacerbate the oxidative damage to proteins, in which PC may be a useful biomarker of OS in CF.

## Graphical abstract

We evaluate the influence of bacterial infection on biomarkers of oxidative stress (catalase, glutathione peroxidase, reduced glutathione), markers of oxidative damage (protein carbonyls, thiobarbituric acid reactive substances), together with the nutritional status and pulmonary function in children with cystic fibrosis. Our results demonstrate that there is a systemic oxidative stress in children with cystic fibrosis. The presence of bacterial infection particularly *Pseudomonas aeruginosa* seems to be determinant to exacerbate the oxidative damage to proteins, in which protein carbonyls may be a useful biomarker of oxidative stress in cystic fibrosis.

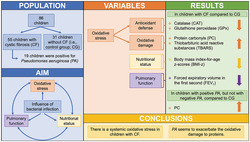

## 1 INTRODUCTION

The overgeneration of reactive oxygen species and the depletion of antioxidants lead to oxidative stress (OS) in cystic fibrosis (CF).^1^ CF is a genetic systemic disease characterized by ion transport abnormalities that causes an increase in the viscosity of secretions and promotes the obstruction and fibrosis of organs.^2^ In the respiratory system, lungs are more susceptible to infection, in which *Staphylococcus aureus* is the first and most common pathogen to infect and colonize the airways of CF patients. Subsequently, lungs may be colonized with *Pseudomonas aeruginosa* and/or *Burkholderia cepacia*.^2^, ^3^, ^4^ Members of *Burkholderia cepacia* complex are very resistant to antibiotic therapy and are associated with the risk of a severe course of the disease*. Pseudomonas aeruginosa* is one of the main causes of chronic pulmonary infection and OS in CF.^2^, ^3^, ^4^ OS can cause pulmonary alterations due to the increase in the respiratory compensation; also, pulmonary infections can lead to the development of malnutrition,^5^ and deficiencies in antioxidant factors in CF patients.^1^, ^6^

The airway infection leads to progressive damage of the lungs, resulting in increased OS and loss of reduced glutathione (GSH), which occurs through its utilization by the inflammatory process.^6^, ^7^ CF transmembrane regulator dysfunction impairs the transport of GSH to the extracellular milieu through the membranes of epithelial cells.^8^ The enzymes catalase (CAT) and glutathione peroxidase (GPx) are also an important part of the antioxidant system. The specific catalytic function of CAT is to decompose hydrogen peroxide into oxygen and water, which is a source of organic peroxides, carbonyl products, and singlet oxygen.^9^ GPx is also able to decompose hydrogen peroxide, to reduce intermediary hydroperoxides in the cyclooxygenase and lipoxygenase tracts, and to decrease the production of prostaglandins and inflammatory leukotrienes.^10^

Given the presence of systemic bacterial infection in CF patients, monitoring the progress of the disease through the clinical status of the individuals together with biomarkers of OS play a fundamental role for its understanding and treatment. In this study, we aimed to evaluate, in a pediatric population with CF, the influence of bacterial infection, including *Pseudomonas aeruginosa*, on biomarkers of OS such as CAT, GPx and GSH, and markers of oxidative damage such as protein carbonyl (PC) and thiobarbituric acid reactive substances (TBARS), together with the nutritional status and lung function in the patients.

## 2 MATERIALS AND METHODS

### 2.1 Study design

This was a cross‐sectional controlled clinical study (April/2009 to December/2011) carried out at the Joana de Gusmão Children's Hospital, Florianópolis/SC ‐ Brazil. The study was approved by the hospital's Human Research Ethics Committee (#048/2008) and registered in the Australian New Zealand Clinical Trials Registry (#ACTRN12611001217998). Written informed consent was acquired by the parents or guardians before participating in the study.

### 2.2 Subjects

A more extensive description of the study population was previously reported.^11^ Briefly, the number of eligible children enrolled in the study during the fixed term (between April 2009 and December 2011) predetermined the sample size. A total of 86 children were initially distributed into two groups, Control Group (CG) and CF Group (CFG). Inclusion criteria for CG were being at the same age and sex as the CFG, normal range for body mass index‐for‐age *z*‐scores (BMI‐z), while exclusion criterion was the presence of CF. Inclusion criteria for CFG were subjects from the Mucoviscidose Support Association of Santa Catarina (Brazil), who were diagnosed with CF by a sweat test (sweat chloride ≥60 mmol/L), and who were 1‐year old or older. Exclusion criteria for both groups were subjects showing fever, diabetes, glucose intolerance, trauma diagnosis, inflammatory disease (asthma, intestinal inflammatory illness, rheumatic illness), degenerative diseases, renal failure, psychiatric disease, cardiovascular disease, and primary or secondary immunodeficiency; and subjects ongoing treatment with antibiotics and/or hormones, anti‐inflammatory, immune suppressant or anti‐histamines drugs, until 30 days before the data collection.

### 2.3 Nutritional status

The weight of children ≤2 years of age was taken using a Filizola®/pediatric‐digital scale (15 kg, 0.01 kg) (Santo André/SP/Brazil), and in children >2 years of age, a Balmak® 50F/digital scale (150 kg, 0.1 kg) (Santa Bárbara do Oeste, SP, Brazil) was used.^12^ The length of children ≤2 years was measured with an infant stadiometer (150 cm, 1 mm) (Sanny®, São Paulo/SP/Brazil), and in children >2 years, the height was verified with an anthropometer (0.1 cm) (Alturaexata®, Belo Horizonte/MG/Brazil).^12^ The nutritional status was classified by BMI.^13^, ^14^

### 2.4 Lung function

Lung function was evaluated only in children >5 years by the forced expiratory volume at the first second (FEV_1_),^15^ using the spirometer Renaissance Spirometry System (Puritan‐Bennett Corporation®, Wilmington/NC/USA). The age (years) in which the disease was diagnosed was also considered.

### 2.5 Bacteriological analysis

Bacteriological analysis was performed in oropharyngeal secretions from subjects after a 10‐hour fast, using a dry swab, which was evaluated microscopically (NIKON‐E200‐microscope‐Chiyoda, Tokyo/Japan) by the Gram method. The presence of pathogenic microorganisms for CF (*Pseudomonas aeruginosa*, *Staphylococcus aureus* and *Burkholderia cepacia*) was assessed by counting colony forming units (CFU).^16^

### 2.6 Biomarkers of oxidative stress

Blood was collected from the cubital region in a vacuum system after 10 h of fasting. To carry out determinations of CAT, GPx, PC, and TBARS, samples were collected in tubes containing ethylene diamine tetraacetic acid (EDTA) as anticoagulant. The separation of plasma and erythrocyte was achieved by centrifugation of whole blood at 2,500 *g* for 10 min. In order to obtain the hemolysates used in CAT and GPx analysis, erythrocytes were washed twice with saline solution and then centrifuged (5000 *g* for 3 min), following successive freezing and thawing procedures. A final centrifugation (5000 *g* for 5 min) supplied the supernatants (hemolysates). Samples were stored at −80°C, except for the plasma samples utilized for TBARS analysis, which were stored in liquid nitrogen (−170°C) until analysis. Aliquots of whole blood were precipitated in trichloroacetic acid (TCA) 12% (1:4, v:v) and stored immediately in liquid nitrogen until GSH analysis. CAT activity was determined by measuring the decrease in a freshly prepared 10mM hydrogen peroxide solution at 240 nm.^17^ GPx was measured in hemolysates at 340 nm,^18^ GSH was measured at 412 nm,^19^ while PC was analyzed at 370 nm.^20^ TBARS were determined using a GBC UV/VIS spectrophotometer (model 916, Sydney/Australia) at 535 nm.^21^ All biochemical parameters were measured in duplicate, except TBARS, which was carried out in triplicate. The chemical substances used in this study were purchased from Sigma‐Aldrich Co. (Ohio/USA).

### 2.7 Statistical analysis

The Kolmogorov–Smirnov test for normality and homogeneity of data was applied. Data are expressed as mean ± standard error of the mean for symmetric distribution and as median and interquartile range (IQR) for asymmetric distribution. Asymmetric variables (CAT, GPx, GSH, and TBARS) were transformed into natural logs before performing multiple linear regression analyses. Multiple linear regression analyses were performed between CG and CFG; between CG and subgroups according to the infection status: CFG negative bacteriology (CFGB−) or CFG positive bacteriology (CFGB+); and according to type of bacterial infection: CFG negative *Pseudomonas aeruginosa* (CFGPa−) or CFG positive *Pseudomonas aeruginosa* (CFGPa+). Sex and age were included as confounding variables in all models. A Statistical Package for the Social Science® for Windows (SPSS‐Inc/2006, Chicago/USA – version 16.0) was used, and a threshold for significance was set at *P* < .05.

## 3 RESULTS

### 3.1 Characteristics of the subjects

The CG included 31 subjects (*n* = 19 female), with a median age of 4.62 years, (IQR: 3.04–8.91) and BMI‐z of 0.30 ± 1.23. During the study period, the CF outpatient unit assisted 75 subjects, in which 20 were excluded from the study due to: age under one year old (*n* = 5), undergoing antibiotic treatment (*n* = 10), showing fever at the time of the study (*n* = 2), presenting a psychiatric disease (*n* = 3). Therefore, the CFG consisted of 55 children (*n* = 29 female) with a median age of 3.89 years (IQR: 1.77–8.52) and BMI‐z of −0.26 ± 1.11. The mean age at diagnosis was 1.9 ± 0.39 years.

Subjects with CF (*n* = 55) were further stratified into subgroups in two different ways. (1) Subjects were stratified according to absence or presence of pathogenic microorganisms in CF (*Pseudomonas aeruginosa*, *Staphylococcus aureus* and *Burkholderia cepacia*): CFGB−, *n* = 27 or CFGB+, *n* = 28. Nineteen subjects had *Pseudomonas aeruginosa* infection, 19 had *Staphylococcus aureus* infection, and three had *Burkholderia cepacian* infection (13 subjects had a combination of two different types of bacterial infection). (2) Subjects were stratified according to the absence or presence of *Pseudomonas aeruginosa*: CFGPa−, *n* = 36 or CFGPa+, *n* = 19.

The BMI‐z for CFG (−0.26 ± 1.11, *P* = .034) and CFGB+ (−0.38 ± 1.28, *P* = .042) were significantly lower compared with CG (0.30 ± 1.23). FEV_1_ was significantly higher in CG (*n* = 14; 88.57 ± 2.67%) compared with CFG (*n* = 21; 66.77 ± 19.33%, *P* = .001). FEV_1_ was significant lower in CFGB+ (*n* = 15; 57.87 ± 17.46, *P* < .001) and CFGPa+ (*n* = 11; 54.82 ± 15.95, *P* < .001) compared with CG (Table 1), even after adjusting for confounding variables (Tables 2, 3, 4).

**TABLE 1:** Participant characteristics and biomarkers of oxidative stress in cystic fibrosis groups and control group

Variables	CG	CFG		CFG bacteriology				CFG *Pseudomonas aeruginosa*			
			*P*	Negative	*P*	Positive	*P*	Negative	*P*	Positive	*P*
	*n* = 31	*n* = 55		*n* = 27		*n* = 28		*n* = 36		*n* = 19	
*Participant characteristics*											
Sex *n* (%F)	19 (61.3)	29 (52.7)	NA	14 (51.9)	NA	15 (53.6)	NA	19 (52.8)	NA	10 (52.6)	NA
Age (year)^a^	4.62 (3.04–8.91)	3.89 (1.77–8.52)	.490	2.82 (1.57–5.34)	**.020**	6.35 (3.49–9.57)	.420	3.38 (1.67–6.11)	.048	6.47 (3.84–0.58)	.220
AD (year)^b^	NA	1.78 ± 3.00	NA	0.88 ± 1.73		2.65 ± 3.67	NA	1.36 ± 2.71	**NA**	2.59 ± 3.40	NA
BMI‐z (*z*‐score)^b^	0.30 ± 1.23	−0.26 ± 1.11	.034	−0.14 ± 0.92	.140	−0.38 ± 1.28	.042	−0.18 ± 0.96	.077	−0.41 ± 1.38	.060
FEV_1_ (%)^b^ ^,^ ^c^	88.57 ± 2.67	66.77 ± 19.33	.001	85.86 ± 10.84	.320	57.87 ± 17.46	**<.001**	78.73 ± 20.63	.440	54.82 ± 15.95	**<.001**
*Biomarkers of oxidative stress*											
CAT (mmol/min/ml)^a^	32.20 (22.34–7.92)	22.57 (17.87–27.14)	.011	24.20 (19.94–7.14)	.042	19.10 (14.72–5.91)	.010	22.68 (19.10–27.14)	.021	18.97 (13.02–27.03)	.019
GPx (μmol/min/ml)^a^	3.25 (2.85–3.48)	2.02 (0.99–3.01)	.004	2.02 (1.38–2.97)	.007	2.05 (0.82–0.06)	.010	1.97 (0.99–2.75)	.001	2.81 (1.04–0.93)	.130
GSH (μmol/ml)^a^	0.78 (0.47–0.96)	0.76 (0.63–0.94)	.560	0.83 (0.66–1.01)	.330	0.69 (0.62–0.90)	.960	0.80 (0.64–0.98)	.530	0.75 (0.62–0.92)	.760
PC (nmol/mg/total protein)^b^	0.038 ± 0.016	0.059 ± 0.042	.081	0.043 ± 0.033	.580	0.073 ± 0.044	.008	0.042 ± 0.027	.700	0.091 ± 0.045	**<.001**
TBARS (nmol/ml)^a^	0.02 (0.01–0.07)	0.21 (0.15–0.25)	**<.001**	0.2 (0.18–0.24)	**<.001**	0.22 (0.18–0.26)	**<.001**	0.21 (0.16–0.24)	**<.001**	0.22 (0.09–0.27)	**<.001**

*Note*: CG: control group; CFG: cystic fibrosis group; %F: percentage of female; AD: age at diagnosis; BMI‐z: body mass index‐for‐age *z*‐score; FEV_1_: forced expiratory volume in the first second; CAT: catalase; GPx: glutathione peroxidase; GSH: reduced glutathione; PC: protein carbonyl; TBARS: thiobarbituric acid reactive substances; NA: not applicable.

^a^ Values are expressed as median (Interquartile range 25–75 percentile).

^b^ Values are expressed as mean ± standard deviation.

^c^ FEV_1_: CG (*n* = 14), CFG (*n* = 21), CFG bacteriology negative (*n* = 6), CFG bacteriology positive (*n* = 15), CFG *Pseudomonas aeruginosa* negative (*n* = 10), CFG *Pseudomonas aeruginosa* positive (*n* = 11)*. P* values of the comparisons between CG versus CFG, CG versus CFG bacteriology (negative and positive), and CG versus CFG *Pseudomonas aeruginosa* (negative and positive) were calculated from independent *t*‐test or Mann–Whitney U test as appropriate. *P* values set in boldface indicate statistical significance (*P* < .05).

**TABLE 2:** Multiple linear regression analysis between control group (*n* = 31) and cystic fibrosis group (*n* = 55)

Variables	Unadjusted		Adjusted	
	β 0 coef. (SEM)	*P*	β 1 coef. (SEM)	*P*
**FEV**_**1**_ (%)	−21.80 (6.07)	.001	−21.74 (5.89)	.001
**CAT** (mmol/min/ml)	−0.30 (0.11)	.007	−0.32 (0.11)	.004
**GPx** (μmol/min/ml)	−0.84 (0.30)	.007	−0.93 (0.30)	.003
**GSH** (μmol/ml)	0.14 (0.12)	.240	0.14 (0.12)	.250
**PC** (nmol/mg/total protein)	0.020 (0.011)	.080	0.023 (0.011)	.048
**TBARS** (nmol/ml)	1.83 (0.17)	≤.001	1.83 (0.17)	≤.001

*Note*: *P* values were calculated from Wald test of the multiple linear regression analysis (95% confidence interval). *P* values set in boldface indicate statistical significance (*P* < .05). β coef.: beta coefficient; SEM: standard error of the mean; β 0 coef.: crude values; β 1 coef.: values adjusted for confounding variables (sex and age); FEV_1_: forced expiratory volume in the first second; CAT: catalase; GPx: glutathione peroxidase; GSH: reduced glutathione; PC: protein carbonyl; TBARS: thiobarbituric acid reactive substances.

**TABLE 3:** Multiple linear regression analysis between control group (*n* = 31) and cystic fibrosis group negative bacteriology (*n* = 27), and between control group and cystic fibrosis group positive bacteriology (*n* = 28)

Variables	Cystic fibrosis group negative bacteriology				Cystic fibrosis group positive bacteriology			
	Unadjusted		Adjusted		Unadjusted		Adjusted	
	β 0 coef. (SEM)	*P*	β 1 coef. (SEM)	*P*	β 0 coef. (SEM)	*P*	β 1 coef. (SEM)	*P*
FEV_1_ (%)	−2.71 (6.72)	.690	−4.09 (6.71)	.550	−30.70 (5.40)	**<.001**	−30.16 (5.38)	**<.001**
CAT (mmol/min/ml)	−0.23 (0.10)	.026	−0.28 (0.11)	.012	−0.37 (0.13)	.007	−0.37 (0.14)	.010
GPx (μmol/min/ml)	−0.751 (0.297)	.016	−0.827 (0.318)	.013	−0.923 (0.342)	.010	−0.990 (0.350)	.007
GSH (μmol/ml)	0.19 (0.17)	.250	0.14 (0.18)	.430	0.09 (0.16)	.560	0.12 (0.17)	.480
PC (nmol/mg/total protein)	0.005 (0.009)	.580	0.007 (0.010)	.510	0.035 (0.012)	.008	0.033 (0.012)	.011
TBARS (nmol/ml)	1.93 (0.20)	≤.001	1.92 (0.21)	≤.001	1.73 (0.22)	≤.001	1.78 (0.22)	≤.001

*Note*: *P* values were calculated from the Wald test of the multiple linear regression analysis (95% confidence interval). *P* values set in boldface indicate statistical significance (*P* < .05). β coef.: beta coefficient; SEM: standard error of the mean; β 0 coef.: crude values; β 1 coef.: values adjusted for confounding variables (sex and age); FEV_1_: forced expiratory volume in the first second; CAT: catalase; GPx: glutathione peroxidase; GSH: reduced glutathione; PC: protein carbonyl; TBARS: thiobarbituric acid reactive substances.

**TABLE 4:** Multiple linear regression analysis between control group (*n* = 31) and cystic fibrosis group negative *Pseudomonas aeruginosa* (*n* = 36), and between control group and cystic fibrosis group positive *Pseudomonas aeruginosa* (*n* = 19)

Variables	Cystic fibrosis group negative *Pseudomonas aeruginosa*				Cystic fibrosis group positive *Pseudomonas aeruginosa*			
	Unadjusted		Adjusted		Unadjusted		Adjusted	
	β 0 coef. (SEM)	*P*	β 1 coef. (SEM)	*P*	β 0 coef. (SEM)	*P*	β 1 coef. (SEM)	*P*
FEV_1_ (%)	−9.84 (6.10)	.120	−10.91 (6.08)	.080	−33.75 (6.10)	**<.001**	−32.68 (6.10)	**<.001**
CAT (mmol/min/ml)	−0.25 (0.10)	.015	−0.29 (0.11)	.008	−0.40 (0.14)	.009	−0.39 (0.15)	.014
GPx (μmol/min/ml)	−0.94 (0.32)	.005	−1.10 (0.33)	.002	−0.64 (0.32)	.060	−0.69 (0.34)	.052
GSH (μmol/ml)	0.15 (0.15)	.300	0.12 (0.15)	.440	0.13 (0.20)	.520	0.17 (0.20)	.410
PC (nmol/mg/total protein)	0.00 (0.01)	0700	0.01 (0.01)	.460	0.05 (0.01)	≤.001	0.05 (0.01)	.001
TBARS (nmol/ml)	1.82 (0.18)	≤.001	1.80 (0.18)	≤.001	1.85 (0.26)	≤.001	1.91 (0.26)	≤.001

*Note*: *P* values were calculated from the Wald test of the multiple linear regression analysis (95% confidence interval). *P* values set in boldface indicate statistical significance (*P* < .05). β coef.: beta coefficient; SEM: standard error of the mean; β 0 coef.: crude values; β 1 coef.: values adjusted for confounding variables (sex and age); FEV_1_: forced expiratory volume in the first second; CAT: catalase; GPx: glutathione peroxidase; GSH: reduced glutathione; PC: protein carbonyl; TBARS: thiobarbituric acid reactive substances.

### 3.2 Biomarkers of oxidative stress in CFG

Compared with CG the mean values obtained for CAT activity (CG = 32.20 vs. CFG = 22.57 mmol/min/ml, *P* = .011), GPx (CG = 3.25 vs. CFG = 2.02 μmol/min/ml, *P* = .004), and TBARS levels (CG = 0.02 vs. CFG = 0.21 nmol/ml, *P* < .001) were significantly different than CFG (Table 1). Even after adjusting for confounding variables (Table 2), the statistical differences remain. No differences were observed regarding the levels of GSH and PC in CFG compared with CG (Table 1); however, after adjusting for confounding variables the PC levels became statistically different between groups (*P* = .048, Table 2).

### 3.3 Biomarkers of oxidative stress in CF subgroups

The biomarker PC, which reflects oxidative damage to proteins, had higher levels in CFGB+ (0.073 nmol/ml, *P* = .008) and CFGPa+ (0.091 nmol/ml, *P* < .001) compared with CG (0.038 nmol/ml) (Table 1), even after adjusting for confounding variables (Tables 3 and 4). No differences in the PC levels were observed between CG and CFGB− (*P* = .510, Table 3), and CG and CFGPa− (*P* = .460, Table 4). GPx activity was lower in CFGB− (2.02 μmol/min/ml, *P* = .007), CFGB+ (2.05 μmol/min/ml, *P* = .010) and CFGPa− (1.97 μmol/min/ml, *P* = .001) compared with CG (3.25 μmol/min/ml, Table 1), even after adjusting for confounding variables (Tables 3 and 4). No differences were observed regarding the GSH levels in CF subgroups compared with CG (Tables 1, 2, 3, 4). CAT activity was significantly lower in all CF subgroups (CFGB− = 24.20 mmol/min/ml, *P* = .042; CFGB+ = 19.10 mmol/min/ml, *P* = .010; CFGPa− = 22.68 mmol/min/ml, *P* = .021; CFGPa+=18.97 mmol/min/ml, *P* = .019) compared with CG (32.20 mmol/min/ml, Table 1), even after adjusting for confounding variables (Tables 3 and 4). Lipoperoxidation measured as TBARS levels were higher in all CF subgroups (all *P* < .001) compared with CG (Table 1), even after adjusting for confounding variables (Tables 3 and 4).

## 4 DISCUSSION

The present study identified higher levels of oxidative damage to proteins and lipids (PC and TBARS, respectively), lower antioxidant defense activities (CAT and GPx), as well as lower lung function (FEV_1_) and nutritional status (BMI‐z) in children with CF. Additionally, the presence of bacterial infections, particularly *Pseudomonas aeruginosa*, worsened the oxidative damage to proteins, nutritional status, and lung function in these subjects. While similar findings have been reported in adults with CF,^1^ this study pioneered early evidence of OS, using a diverse set of blood biomarkers, in association with bacterial infections in childhood with CF.

These findings strongly indicate structural and functional oxidative damage^3^, ^6^, ^22^ as well as a decrease in the antioxidant defenses suggesting a systemic OS in individuals with CF.^23^, ^24^ Similar to our study, oxidative damage to proteins was also observed in 23 children with CF, in which PC levels, obtained through the bronchoalveolar lavage samples, were more than two fold higher in CF (0.098 nmol/mg) compared with a healthy CG (*n* = 7, 0.040 nmol/mg, *P* < .001).^22^ The increase in TBARS levels obtained in our study resemble those obtained in a similar study^6^ that used malondialdehyde (MDA), as a more specific marker of oxidative damage to lipids. The majority of 70 pediatric subjects with CF (age range: 1–18 years) in stable clinical condition presented with elevated MDA levels despite normal plasma vitamin E, A, and C.^6^ Another study also found increased MDA levels in 40 adults with CF in stable clinical conditions (176.1 ± 15.9 nmol/L, *P* < .05) compared with 25 healthy control subjects (129.6 ± 12.9 nmol/L).^25^ Contrary to these results, a large study conducted by Lagrange‐Puget and colleagues^26^ identified decreased levels of MDA and TBARS in 312 subjects with CF compared with the 53 controls, suggesting a depletion in fatty acids. The increase in fatty acid oxidation induced by reactive oxygen species generation may be causing a fatty acid deficiency.^26^ A hallmark of CF disease is the imbalance in polyunsaturated fatty acids (PUFAs) with low levels of the anti‐inflammatory docosahexaenoic acid and high levels of arachidonic acid. This imbalance in PUFAs may contribute to the inflammatory status observed in CF.^27^ Wojewodka and colleagues^27^ conducted a prospective cohort study assessing lipid peroxidation by indirectly measuring TBARS as well as PUFAs profile in 53 adults with CF. Out of 53 subjects, 37 experienced pulmonary exacerbations during the 24‐month study period, and 13 subjects were closely followed until the end of pulmonary exacerbation. The authors found that high levels of docosahexaenoic acid may contribute to the resolution of lipid peroxidation.^27^

Regarding the enzymatic antioxidant defenses, our findings showed that CAT and GPx activity were lower in subjects with CF compared with controls. A similar CAT response was found in another large study conducted in 100 pediatric subjects with CF (age range: 1–18 years), compared with 25 healthy controls with same age range, indicating the increased susceptibility of erythrocytes to OS in CF.^23^ Other studies also revealed that OS remained high regardless of the symptomatic treatment.^6^, ^26^ The decrease in GPx activity in plasma was also found in a study with 27 subjects with CF (age range: 7–20 years, 263.6 ± 42 U/L, *P* < .05) compared with 17 healthy controls (296.9 ± 57 U/L), although the erythrocytes levels were not different between groups.^24^ This decrease in GPx activity may be due to, besides the depletion of its co‐factor GSH, CF‐related selenium deficiency, which even after supplementation, subjects with CF showed only marginal levels of selenium.^28^

Concerning the findings obtained for the endogenous and ubiquitous antioxidant GSH,^9^ which revealed no statistical differences among the CF groups, it was not surprising, considering the chronic disease condition of the children, who were examined at least after one year of diagnosis (Table 1). GSH is an important first front and generalist antioxidant, and it usually present at high molar concentrations, also acting as a co‐factor for two important antioxidant enzymes GPx and GST.^9^ GSH was probably persistently depleted by the continuous oxidative challenge promoted by the inflammatory process associated with CF, as found in other related study assessing GSH levels in patients with Down syndrome.^29^

Additionally, subjects with CF showed lower FEV_1_ and BMI‐z compared with controls. It is suggested that resting energy expenditure is increased by 7–35% in subjects with CF, which may contribute to catabolism and lung function deterioration.^30^ A population‐based cohort study with 909 adults with CF demonstrated that those with lower BMI displayed a worse lung function, and improvements in lung function were observed with an increased in BMI within the underweight group.^5^ Therefore, nutritional status is directly associated with lung function in CF patients.

It has been postulated that bacterial infection of the airways may contribute to dysregulation of redox balance in CF. Because subjects participating in this study were categorized based on bacterial colonization, the effect of colonization could be further estimated. We demonstrated that bacterial infections worsened the oxidative damage to proteins, nutritional status, and lung function of subjects with CF by showing increased levels of PC, and decreased FEV_1_ and BMI‐z. These findings are in agreement with a previous study which demonstrated elevated PC levels in *Pseudomonas aeruginosa* and *Staphylococcus aureus* infected CF pediatric subjects (1.9 ± 0.64, and 1.87 ± 0.45 nmol/mg protein) versus controls (0.94 ± 0.19 nmol/mg protein; 𝑃 < .05).^3^ Other biomarkers of OS assessed in this present study had similar responses in subjects with CF compared with controls regardless the presence or absence of *Pseudomonas aeruginosa* and/or other pathogens.

The limitations of this study should be considered when interpreting the results. Although our sample size was similar to others using comparable methods, it is still relatively restricted. Therefore, it is possible that the number of participants may have led to failure to detect other meaningful outcomes. Additionally, the recruitment took place at a single referral center for CF treatment, which could also limit our findings. The study was of cross‐sectional design preventing to derive causal relationships among variables. Notably, this study used a diverse set of blood biomarkers to characterize OS in CF, which are readily available from individuals of any age and with any disease severity.

In conclusion, the present study examined various clinical aspects of the CF disease such as the presence of infection by pathogenic bacteria, lung function, and different ways of grouping individuals. The decrease detected in GPx and CAT activity together with an increase in PC and TBARS contents as well as the apparent inability to maintain higher GSH levels clearly indicate an early evidence of an altered redox response in children with CF. Such chronic oxidative insult associated with bacterial infection particularly *Pseudomonas aeruginosa* seems to exacerbate the oxidative damage to proteins and to contribute to progressive lung disease and nutritional status depletion. In this regard, PC may be a useful marker of oxidative modifications of plasma proteins in CF.

## CONFLICT OF INTEREST

The authors have declared that there is no conflict of interest.

## ETHICS STATEMENT

The study was approved by the hospital's Human Research Ethics Committee (#048/2008), and written informed consent was acquired by the parents or guardians before participating in the study.

## AUTHOR CONTRIBUTIONS

Conceptualization: GDB and EAMM. Data curation: EAM and YAFM. Formal analysis: GDB, LCRP, and MBF. Investigation: GDB and LCRP. Methodology: GDB, EAMM, LCRP, DLO, JCV, EBP, EBSM, EB, and NLN. Project administration, Resources, Software and Supervision: EAMM. Validation: GDB, YMFM, and EAMM. Roles/Writing ‐ original draft: GDB. Writing ‐ review & editing: LCRP, MBF, EAMM, YMFM, and DWF.

## CLINICAL TRIAL REGISTRATION

Australian New Zealand Clinical Trials Registry (#ACTRN12611001217998).

## Data Availability

The data that support the findings of this study are available from the corresponding author upon reasonable request.
